# The Inhibition of Ocean Acidification on the Formation of Oyster Calcified Shell by Regulating the Expression of *Cg*chs1 and *Cg*chit4

**DOI:** 10.3389/fphys.2019.01034

**Published:** 2019-08-13

**Authors:** Yukun Zhang, Zhaoqun Liu, Xiaorui Song, Shu Huang, Lingling Wang, Linsheng Song

**Affiliations:** ^1^Liaoning Key Laboratory of Marine Animal Immunology, Dalian Ocean University, Dalian, China; ^2^Liaoning Key Laboratory of Marine Animal Immunology and Disease Control, Dalian Ocean University, Dalian, China; ^3^Dalian Key Laboratory of Marine Animal Disease Prevention and Control, Dalian Ocean University, Dalian, China; ^4^Functional Laboratory for Marine Fisheries Science and Food Production Processes, Qingdao National Laboratory for Marine Science and Technology, Qingdao, China

**Keywords:** ocean acidification, chitinase, chitin synthetase, shell formation, oyster larvae

## Abstract

The biosynthesis of a calcified shell is critical for the development of oyster larvae. This process can be severely inhibited by CO_2_-induced ocean acidification, causing mass mortality of oyster larvae. However, the underlying molecular mechanism of such process has not been well explored until now. In the present study, a homolog of chitin synthase (named as *Cg*chs1) and a homolog of chitinase (named as *Cg*chit4) were identified from the Pacific oyster *Crassostrea gigas*. The cDNA sequences of *Cg*chs1 and *Cg*chit4 were of 813 bp and 2118 bp, encoding a putative polypeptide of 271 amino acids and 706 amino acids, respectively. There were a Chitin_synth_2 domain and a Glyco_18 domain in the inferred amino acid sequences of *Cg*chs1 and *Cg*chit4, respectively. Both *Cg*chs1 and *Cg*chit4 shared high sequence identity with their homologs in vertebrates. In addition, when oyster larvae were exposed to acidification treatment (pH 7.4), their shell biosynthesis process was seriously restrained. The expression level of *Cg*chs1 mRNA was significantly suppressed while that of *Cg*chit4 was dramatically activated upon acidification treatment. *Cg*chs1 and *Cg*chit4 are critical enzymes for chitin metabolism, and such changes in their mRNA expression could result in the decrease of chitin content in oyster larvae’s shells, which might lead to the failure of shell formation. Therefore, results in the present study suggested that acidified seawater might inhibit the formation of oyster calcified shell by suppressing the biosynthesis of chitin.

## Introduction

Marine molluscs, such as oysters and scallops, secrete calcified shells as a supporting frame for their bodies and for the protection from predators (Zhang et al., 2012; Hendriks et al., 2015). The biosynthesis of the larval shells is originated from a peculiar group of ectodermal cells in the early developmental stages of embryos, which are called shell gland (Bevelander and Nakahara, 1969; Kniprath, 1979). When the ectodermal cells are sunken to construct the shell gland, the surrounding cells begin to generate the outmost matrix shell layer, the periostracum (Eyster and Morse, 1984). The cells of shell gland region are then turned over from inside to outside, transforming into the mantle epithelium of larvae (Kniprath, 1979; Bielefeld and Becker, 1991). During this process, the periostracum spans the whole shell field epithelial surface, and only then, the crystalline calcium carbonate appears for the first time (Eyster, 1986). The onset of shell mineralization occurs during the trochophore larval stage, which is usually about 20 h post fertilization (Kniprath, 1981; Nudelman et al., 2006), and the shell is called the prodissoconch I (PDI) (Weiss et al., 2002). The bivalve PDI is enlarged until the embryo is entirely enfolded and able to close its two valves. Transformation into the motile D-shape larvae then occurs. The D-shape larvae enlarges PDI to form prodissoconch II (PDII) which is fully calcified (Carriker and Palmer, 1979; Waller, 1981).

In the past few decades, these crucial processes have been severely inhibited by ocean acidification (OA) (Orr et al., 2005; Waldbusser and Salisbury, 2014). The oceans absorb the excess CO_2_, and cause changes in oceanic chemistry, including increased levels of dissolved CO_2_, a reduction in pH from pre-industrial levels of 8.2 to a projected 7.8 in 2100, and a reduction in the saturation state of biologically useful forms of CaCO_3_, especially aragonite and calcite (Sabine et al., 2004; Kerr, 2010). Compared with the adults, molluscan larvae are especially sensitive to OA during the hours to days-long bottleneck when initial shell (PDI) is formed during embryogenesis (Waldbusser et al., 2013; Talmage and Gobler, 2009). During calcification of PDI, the calcification surfaces are in greater contact with ambient seawater than during the following shell stages (Waldbusser et al., 2014). Before PDI shell formation, the larvae mainly depend on the maternal energy from eggs for survival. Failure of forming complete shell before exhausting the maternal energy reserves would result in eventual mortality of larvae (Barton et al., 2012). Therefore, a better understanding of how OA suppresses the formation of calcified shell during larval development of oysters will not only contribute to illustrate the stress response patterns of marine calcifiers upon OA, but also provide new insights into the shellfish ecological aquaculture industry in a fast-changing environment.

Chitin synthase and chitinase, the crucial enzymes for the metabolism of chitin, have been proved to be important in shell biosynthesis of bivalve larvae because the calcified shell is deposited at early D-shape larvae stage to cover the chitin shell of trochophore larvae (Weiss and Schonitzer, 2006; Zhang et al., 2012). For example, a homologous gene for chitin synthetase was identified from mussel *Mytilus galloprovincialis*, which was expressed in the early embryo and the shell forming tissue of larvae (Weiss et al., 2006). It was speculated that chitin synthesis contributed via signal transduction pathways to the implementation of hierarchical patterns into chitin mineral-composites such as prismatic, nacre, and crossed-lamellar shell types (Schonitzer and Weiss, 2007). Meanwhile, it was found that the chitinase of pearl oyster *Pinctada fucata* might possess key function in the biomineralization of shell (Li et al., 2017). Similar results were also reported in *C. gigas* that chitinase could regulate the shell formation in larvae under the modulation of dopamine (Liu et al., 2018). However, the correlation between OA and the expressions of chitinase and chitin synthetase has never been reported during the formation of calcified shell in oyster larvae.

In the present study, a chitinase (*Cg*chit4) and a chitin synthase (*Cg*chs1) were identified from the Pacific oyster *C. gigas* with the major objectives to (1) explore the alteration of their expression levels during larval development stages as well as under acidification treatments, (2) illustrate their involvements in the biosynthesis of calcified shell during larval development, and (3) reveal the underlying mechanisms of how acidified seawater influence the formation of oyster calcified shell.

## Materials and Methods

### Oyster Larvae, Acidification Treatment, and Sample Collection

The Pacific oysters *C. gigas* (about 2-year old, averaging 150 mm in shell length) were collected from a local breeding farm in Dalian, Liaoning Province, China, and maintained in the aerated seawater at 20°C for 14 days before processing. Eggs and sperms were scraped from female and male individuals, respectively, and mixed together in a small tank of 10 L for fertilization. The fertilized eggs were observed with a microscope to see if the first polar body was formed at 15 min after fertilized. The fertilized eggs were observed again to see if the second polar body was formed at 90 min post fertilization. Then, the fertilized eggs were transferred into a larger tank containing 100 L of aerated seawater at 20°C. Sample collection began at 12 h post fertilization (hpf) according to previous description (Kin et al., 2009; Zhou et al., 2012). Trochophore (12 hpf), early D-shape larvae (17 hpf), and D-shape larvae (24 hpf) were authenticated through microscopy and collected.

Trochophores collected at 12 hpf were equally divided into three groups (three replicates for each group). Larvae without any treatment were designated as the control group (pH = 8.10 ± 0.05), while those in the moderate CO_2_ treatment group (pH 7.8 group) were bathed in acidified seawater (pH = 7.80 ± 0.05). In the severe CO_2_ treatment group (pH 7.4 group), the trochophore larvae were bathed in acidified seawater with pH value of (7.40 ± 0.05). The pH value of the CO_2_ exposure group was controlled using an acidometer (AiKB, Qingdao, China). Total alkalinity was measured by end-point titration of 25 mmol L^–1^ HCl on 50 mL samples. The total alkalinity in the pH 8.1, pH 7.8, and pH 7.4 groups was 2849.1 ± 14.5 μeq kg^–1^, 2416.3 ± 125.1 μeq kg^–1^ and 2110.6 ± 75.3 μeq kg^–1^, respectively. And the partial pressure of CO_2_ was about 658.1 ± 11.0 ppm, 1217.3 ± 11.6 ppm and 2268.4 ± 50.1 ppm in pH 8.1, pH 7.8, and pH 7.4 treatment groups, respectively, (Table 1). Larvae in the three groups were sampled at 12 h after treatment (at D-shape larvae stage). Each sample was added into 1 mL of TRIzol reagent in 1.5 mL EP tube for RNA isolations (Invitrogen, United States).

**TABLE 1:** Parameters of the seawater in the present study.

**pH control**	**pCO_2_**	**Total alkalinity**
8.10 ± 0.05	658.1 ± 11.0 ppm	2849.1 ± 14.5 μeq kg^–1^
7.80 ± 0.05	1217.3 ± 11.6 ppm	2416.3 ± 125.1 μeq kg^–1^
7.40 ± 0.05	2268.4 ± 50.1 ppm	2110.6 ± 75.3 μeq kg^–1^

Larvae for whole-mount *in situ* hybridization (WMISH) were first narcotized by gradual addition of 7.5% MgCl_2_, followed by fixation in fresh 4% paraformaldehyde (PFA) in 0.01 M phosphate buffer saline (PBS) (0.2 g KCL, 8.0 g NaCl, 2.9 g Na_2_HPO_4_.12H_2_O, 0.2 g KH_2_PO_4_, pH 7.40) at 4°C for 3 h and washed three times (15 min for each time) with pre-cold PBS. After fixation, the samples were dehydrated and stored in pure methanol at −20°C for the subsequent WMISH. For the scanning electron microscopy (SEM) analysis, oyster larvae were pre-fixed in 4% PFA before the addition of 5% glutaraldehyde and incubated at 24°C for 4 h. The samples were rinsed twice with 0.1 M cacodylate and dehydrated with graded acetone.

### Total RNA Extraction and cDNA Synthesis

Total RNA was isolated from the oyster larvae using TRIzol reagent according to the standard protocol. The RNA concentration was measured on a NanoDrop 2000 reader (Thermo Fisher, United States), and the integrity and purity of RNA were examined by electrophoresis running in 1.0% agarose gel. The total RNA was then treated with DNaseI (Takara, China) to remove trace DNA contamination. The synthesis of the first-strand cDNA was carried out with Promega M-MLV RT with oligo (dT)-adaptor priming according to the manufactory’s protocol. The synthesis reaction was performed at 42°C for 1 h, terminated by heating at 95°C for 5 min. The synthesis reaction product of cDNA was stored at −80°C for the subsequent SYBR Green fluorescent quantitative real-time PCR.

### Gene Cloning and Sequence Analysis

Blastp analysis of all oyster protein sequences revealed that one sequence (CGI_10024867) was homologous to chitinase identified previously, and one sequence (CGI_10025283) was homologous to chitin synthase. The open reading frame (ORF) of *Cg*chs1 and *Cg*chit4 were cloned from cDNA library using specific primers (P1, P2 and P3, P4, Table 2). The homology searches of the cDNA sequences and protein sequences of *Cg*chs1 and *Cg*chit4 were conducted with BLAST algorithm at the National Center for Biotechnology Information (NCBI)^1^. The deduced amino acid sequence was analyzed with the Expert Protein Analysis System^2^. The protein domain was predicted with the simple modular architecture research tool (SMART) version 5.1^3^. Multiple sequence alignment of the *Cg*chs1 and *Cg*chit4 with other chitinases and chitin synthetases was created by the ClustalW multiple alignment program^4^ and multiple sequence alignment show program^5^.

**TABLE 2:** Sequences of the primers used in this study.

**Primer**	**Sequence (5′–3′)**
**Clone primers**	
P1 (*Cg*chs1-F)	CCAAATGAGTTCGTGGCTGA
P2 (*Cg*chs1-R)	AAGACAAGATAACCTGAGGGGAT
P3 (*Cg*chi4-F)	TCTCTAACGGACTCTATTTATGCG
P4 (*Cg*chit4-R)	TTTCCACAATTTCAATGCCA
**RT-PCR primers**	
P5 (*Cg*chs1-RT-F)	AAGCCTCACACTTTACCAGAA
P6 (*Cg*chs1-RT-R)	TTGATACCAGACAATCGGACC
P7 (*Cg*chit4-RT-F)	AACCCCGCCACCCCTAC
P8 (*Cg*chit4-RT-R)	TATCCCGTTGCTCCGTTATCA
P9 (*Cg*-EF-F)	AGTCACCAAGGCTGCACAGAAAG
P10 (*Cg*-EF-R)	TCCGACGTATTTCTTTGCGATGT
**WEISH primer**	
P11 (*Cg*chs1-WEISH-F1)	CCCCTCAGGTTATCTTGTC
P12 (*Cg*chs1-WEISH-R1)	CTGGGAAACTGTTGAATGT
P13 (*Cg*chs1-WEISH-F2)	CCGTAATACGACTCACTATAGCCC
	CTCAGGTTATCTTGTC
P14 (*Cg*chs1-WEISH-R2)	CGGATTTAGGTGACACTATAGCTG
	GGAAACTGTTGAATGT
P15 (*Cg*chit4-WEISH-F1)	AACTCGTGGACCAGGC
P16 (*Cg*chiti4-WEISH-R1)	ATAAGGTACGCTTTGCTCT
P17 (*Cg*chit4-WEISH-F2)	CCGTAATACGACTCACTATAGAAC
	TCGTGGACCAGGC
P18 (*Cg*chit4-WEISH-R2)	CGGATTTAGGTGACACTATAGATAA
	GGTACGCTTTGCTCT

### Scanning Electron Microscope

Oyster larvae were collected and immersed in 2.5% glutaraldehyde at 4°C overnight, and then resuspended twice with 0.1 M methyl arsenate. After dehydrated in an ascending series of acetone, the larvae samples were coated with gold atoms, and their morphology was observed through scanning electron microscope at a voltage of 20–25 kv.

### Quantitative Real-Time PCR

The expression levels of *Cg*chs1 and *Cg*chit4 in different developmental stages were measured by an ABI PRISM 7500 Sequence Detection System with a total volume of 25.0 μL, containing 12.5 μL of SYBR Green Mix (Takara), 0.5 μL of each primers (10 μmol L^–1^) (P5, P6 and P7, P8, Table 2), 2.0 μL of cDNA, and 9.5 μL of DEPC-water. The oyster EF (elongation factor) was used as internal control (Table 2). Dissociation curve analysis of amplification products was performed to confirm that only one PCR product was amplified and detected. The comparative average cycle threshold method was used to analyze the mRNA expression level of the immune-related genes according to Zhang et al. (2008). All data were given in terms of relative mRNA expression using the 2^–ΔΔ*Ct*^ method (Livak and Schmittgen, 2001).

### Whole-Mount *in situ* Hybridization

Eight probe primers (Table 2) were used to synthesize the RNA probe. The paired primers, P11 and P12, P15 and P16, were used to amplify the double-stranded DNA probe of chitin synthetase and chitinase, respectively. The T7 promoter sequence was added to the 5′ end of P13 and P17 primer, while the SP6 promoter sequence was added to 5′ end of P14 and P18. The double-stranded DNA probe with the T7/SP6 promoter sequence primer was amplified in order to add the promoter sequence for the *in vitro* transcription of single stranded RNA probe with T7/SP6 RNA polymerase and digoxin DIG RNA Labeling Mixture (Roche, Switzerland). The detailed step was conducted following the previously research (Thisse and Thisse, 2008).

### Statistical Analysis

All the data were shown as mean ± S.D. The two-sample student’s tests were performed for the comparisons between groups. Multiple group comparisons were executed by one-way ANOVA and followed by a Turkey multiple group comparison tests using Statistical Package for Social Sciences (SPSS) 16.0 Software. The difference was considered as significant at *p* < 0.05.

## Results

### Sequence Features and Phylogenetic Relationship Constructs of *Cg*chs1 and *Cg*chit4

**FIGURE 1:**
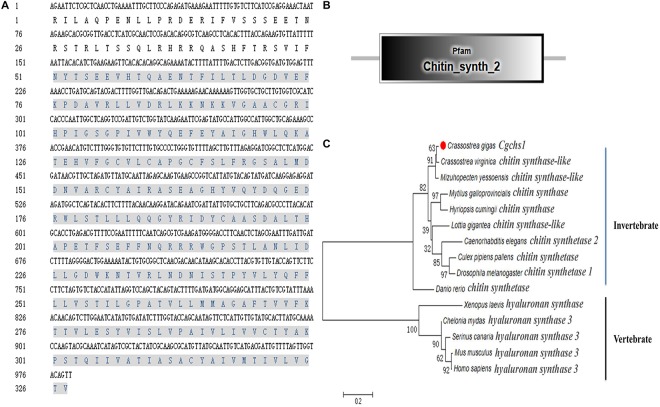
Domain characters of *Cg*chs1. **(A)** Complete nucleotide and deduced amino acid sequence of *Cg*chs1. The Chitin_synth_2 region is in gray. **(B)** Protein domains of *Cg*chs1 were predicted by SMART (http://smart.embl.de/). **(C)** Phylogenetic relationship of chitin synthase protein sequences. Mega 6.0 program was used to construct the neighbor-joining (NJ) tree, algorithm based on the multiple sequence alignment of chitin synthase protein sequences. Chitin synthase protein from different species: *Crassostrea gigas* (XP_011422035.1); *Crassostrea virginica* (XP_022329201.1); *Mizuhopecten yessoensis* (XP_021358360.1); *Culex pipiens pallens* (XP_001866798.1); *Caenorhabditis elegans* (NP_001300545.1); *Drosophila melanogaster* (AAG09735.1); *Homo sapiens* (AAK73797.1); *Mus musculus* (NP_032243.2); *Chelonia mydas* (XP_007062507.1); *Serinus canaria* (XP_009089183.1); *Danio rerio* (AJW72838.1); *Mytilus galloprovincialis* (ABQ08059.1); *Lottia gigantea* (XP_009047936.1); *Hyriopsis cumingii* (ALJ53301.1); *Xenopus laevis* (AAC60343.1).

**FIGURE 2:**
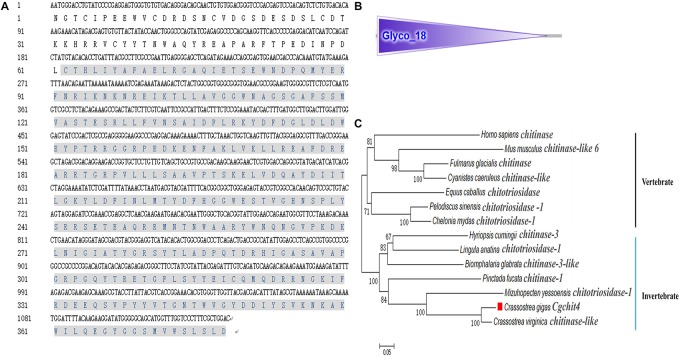
Domain characters of *Cg*chit4. **(A)** Complete nucleotide and deduced amino acid sequence of *Cg*chit4. The Glyco_18 domain is in gray. **(B)** Protein domains of *Cg*chit4 were predicted by SMART (http://smart.embl.de/). **(C)** Phylogenetic relationship of chitinase protein sequences. Mega 6.0 program was used to construct the neighbor-joining (NJ) tree, algorithm based on the multiple sequence alignment of chitinase protein sequences. Chitinase protein from different species: *C. gigas* (EKC36140.1); *Crassostrea virginica* (XP_022315646.1); *M. yessoensis* (OWF49953.1); *Lingula anatina* (XP_013402309.1); *Hyriopsis cumingii* (AFO53261.1); *Biomphalaria glabrata* (XP_013090777.1); *Equus caballus* (ABX82529.1); *Pinctada fucata* (ANJ60740.1); *H. sapiens* (AAB04534.1); *Pelodiscus sinensis* (XP_014434032.1); *C. mydas* (EMP30858.1); *Cyanistes caeruleus* (XP_023797990.1); *Fulmarus glacialis* (XP_009571995.1); *Mus musculus* (NP_848499.1).

A potential oyster chitin synthase was screened out from the NCBI database with the GenBank accession number of XP_011422035.1 (Figure 1A). In the present study, an 813 bp cDNA fragment of *Cg*chs1 was amplified, which encoded a presumptive peptide of 271 amino acids. The predicted molecular weight of the derived *Cg*chs1 protein was 30.16 kDa with a Chitin_synth_2 domain (from Glu872 to Val1143) (Figure 1B) and its theoretical isoelectric point (pI) was 6.08. Meanwhile, a potential oyster chitinase (*Cg*chit4) with the GenBank accession NO. XP_011432127.1 was also annotated from oyster genome. A cDNA fragment of 2118 bp was amplified, which encoded Cgchit4 (706 amino acids) with the theoretical molecular weight of 80.43 kDa (Figure 2A). The theoretical pI of *Cg*chit4 was 9.13 which predicted by Compute pI/Mw tool of ExPasy. There was a Glyco_18 domain (from His81 to Asp426) found in *Cg*chit4 (Figure 2B).

**FIGURE 3:**
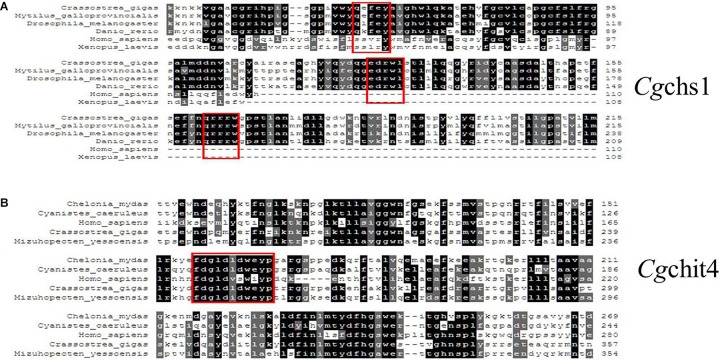
**(A)** Multiple sequence alignment of the *Cg*chs1 and *Cg*chit4 proteins. Multiple sequence alignment of *Cg*chs1 with chitin synthase from other species: *C. gigas* (XP_011422035.1); *M. galloprovincialis* (ABQ08059.1); *Drosophila melanogaster* (AAG09735.1); *Danio rerio* (AJW72838.1); *H. sapiens* (AAK73797.1); *Xenopus laevis* (AAC60343.1). **(B)** Multiple sequence alignment of *Cg*chit4 with known chitinases from other species: *C. mydas* (EMP30858.1); *C. caeruleus* (XP_023797990.1); *H. sapiens* (AAB04534.1); *C. gigas* (EKC36140.1); *M. yessoensis* (OWF49953.1). Red boxes showed the active site of FDGLDXDW in the Glyco_18 domain of *Cg*chit4.

The phylogenic trees were constructed by using the amino acid alignment of *Cg*chs1 and *Cg*chit4 (Figures 1C, 2C). There were two major distinct branches for the chs1 and chit4 from vertebrate and invertebrate. The chitin synthase and chitinase from vertebrates were clustered together as a sister branch from invertebrate ones. Both *Cg*chs1 and *Cg*chit4 were primarily aggregated with their homologs from *C. virginica* and *M. yessoensis*, and then clustered with those from other invertebrate species (Figures 1C, 2C). The amino acid sequence of *Cg*chs1 shared 44% identity with chitin synthase from *M. galloprovincialis*, 43% identity with that from *Drosophila melanogaster*, 41% identity with that from *D. rerio*, 24% identity with that from *X. laevis*, and 22% identity with that from *Homo sapiens*. Multiple alignment analysis found that there were three motifs including QXXEY, EDRXL, and QXRRW in the Chitin_synth_2 domain of *Cg*chs1 (Figure 3A). The amino acid sequence of *Cg*chit4 shared 52% identity with chitinase from *Mizuhopecten yessoensis*, 51% identity with that from *Chelonia mydas*, 47% identity with that from *Cyanistes caeruleus*, and 41% identity with that from *H. sapiens*. An active site of FDGLDXDW was identified in the Glyco_18 domain of *Cg*chit4, which was similar with their homologs from vertebrate species (Figure 3B).

### The mRNA Expression Levels of *Cg*chs1 and *Cg*chit4 in Trochophore, Early D-Shape Larvae and D-Shape Larvae in Response to Acidification Treatment

**FIGURE 4:**
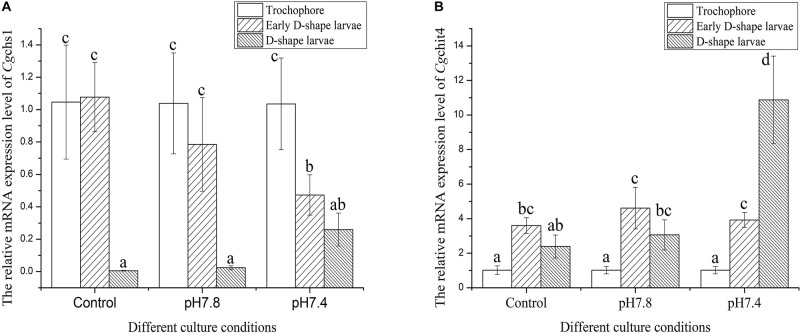
The expression levels of *Cg*chs1 and *Cg*chit4 mRNA transcripts in trochophore, early D-shape larvae and D-shape larvae under different acidification treatment. *Cg*chs1 and *Cg*chit4 mRNA expression levels in different development stage and in different culture conditions of oyster *C. gigas* were detected by qRT-PCR. **(A)** *Cg*chs1 and *Cg*chit4 transcript levels in trochophore larvae, early D-veliger larvae and D-veliger larvae of three acidification treatment (control, pH 7.8, and pH 7.4) were normalized to that of in trochophore larvae, respectively. **(B)** Vertical bars represent the mean ± S. D. (*N* = 3), and the letters (a,b,c) were used to present significant differences.

The expression levels of *Cg*chs1 and *Cg*chit4 mRNA were investigated in trochophore, early D-shape larvae and D-shape larvae in response to acidification treatment by quantitative real-time PCR. In the control group, the expression level of *Cg*chs1 mRNA at D-shape larvae stage was significantly lower comparing with that at trochophore and early D-shape larvae stages (Figure 4A, *p* < 0.05), nevertheless the expression level of *Cg*chit4 mRNA in early D-shape larvae was obviously higher than that in trochophore and D-shape larvae (Figure 4B, *p* < 0.05). No distinct change of the mRNA expressions of *Cg*chs1 and *Cg*chit4 was observed after moderate acidification treatment (pH 7.8), while the expression level of *Cg*chs1 mRNA (Figure 4A, *p* < 0.05) was significantly decreased and that of *Cg*chit4 mRNA (Figure 4B, *p* < 0.05) was significantly increased after the severe acidification treatment (pH 7.4).

### Morphologic Observation of Oyster Larvae Shell Under Acidification Treatment

**FIGURE 5:**
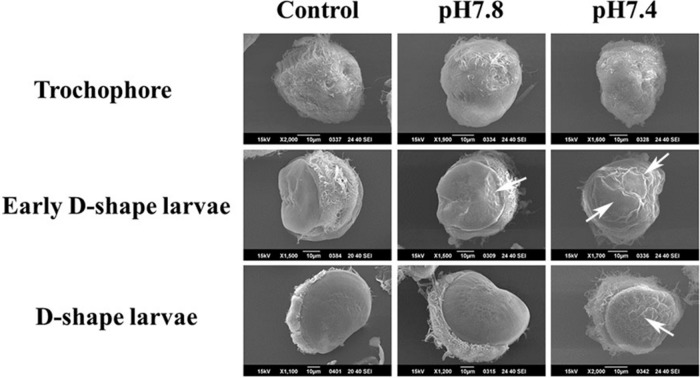
Morphological characteristics of oyster larvae under acidification treatment. The oyster larvae from three different development stages treated with three different acidification treatments, respectively, were collected and observed by scanning electron microscope. Trochophore, Early D-shape larvae and D-shape larvae were employed for the acidification treatments, and they were divided into three different acidification (Control, pH 7.8, and pH 7.4) groups, respectively. The white arrow showed the predicted location of shell malformation.

Scanning electron microscopy was used for inspecting the morphological characteristics of oyster larvae under the different acidification treatments (Figure 5). A uniform and smooth shell appeared in oyster larvae from trochophore to D-shape larvae in the control group, exhibiting a normal developmental process. The sizes of larvae in control group and moderate acidification treatment were almost same, while the initial shell formation of larvae could be delayed in severe acidification treatment group. As shown in Figure 5, there was a small concave on the ventral edge of the initial shell of early D-shape larvae in the moderate acidification group (indicated by the white arrow). The initial shell of early D-shape larvae in the severe acidification group was not as smooth as that in the control group, and the shell length was also shorter (indicated by the white arrows). Besides, the initial shell of D-shape larvae in the severe acidification treatment group was full of wrinkles and the calcified layer was barely formed comparing with that in the control group (indicated by the while arrow).

### The Distribution of *Cg*chs1 and *Cg*chit4 mRNA Transcripts in Trochophore, Early D-Shape Larvae and D-Shape Larvae Under Acidification Treatment

**FIGURE 6:**
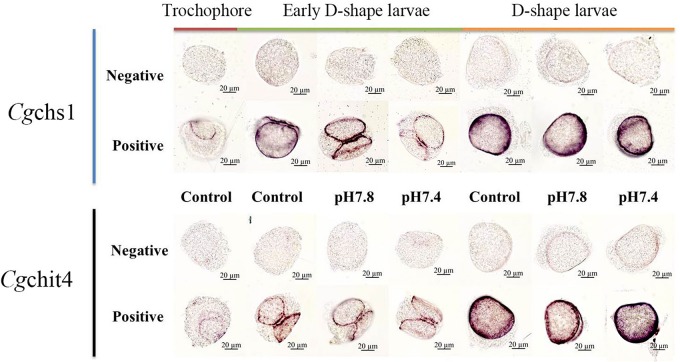
The mRNA distributions of Cgchs1 and Cgchit4 in trochophore, early D-shape larvae and D-shape larvae under acidification treatment. The mRNA location of *Cg*chs1 and *Cg*chit4 were investigated via WMISH. Every gene was carried out with a negative probe test and a positive probe test, respectively. Trochophore larvae were initially cultured at normal seawater and sampled as control. Trochophore larvae were then divided into three groups (control, pH 7.8, and pH 7.4 groups) for the further development and acidification treatment. Early D-shape larvae and D-shape larvae were collected under acidification treatment.

The localization of *Cg*chs1 and *Cg*chit4 mRNA transcripts in trochophore, early D-shape larvae and D-shape larvae under acidification treatment were investigated via WMISH. The positive signals were dyed in bluish violet, and they were mainly distributed on the edge of the newly formed shells. In both the control and acidification treatment trochophore larvae, the positive signal was mainly located on the margin of shell field in. And in D-shape larvae, the positive signal was found on the edge of periostracum. No specific signals were inspected in all over the negative control groups, suggesting the specificity of the antisense probes and the creditability of the current results (Figure 6).

## Discussion

As the exoskeletons for most molluscan species, shells have been extensively studied in both adults and larvae for their evolutionary and economic importance. The larval shell emerges early in embryogenesis of molluscs, and chitin biogenesis is crucial for this process (Peters, 1972). The ongoing CO_2_-induced ocean acidification has been reported to severely threaten the shell formation of molluscan larvae and cause mass mortality (Kurihara et al., 2007; Miller et al., 2009; Kroeker et al., 2010; Talmage and Gobler, 2010; Parker et al., 2012). Previous studies have already demonstrated that the early development of bivalves including oyster *C. gigas* (Kurihara et al., 2007; Barros et al., 2013), blue mussel *Mytilus edulis* (Gazeau et al., 2010) and barnacle *Amphibalanus amphitrite* (McDonald et al., 2009) is negatively affected by acidified conditions. In the present study, a chitin synthase (*Cg*chs1) and a chitinase (*Cg*chit4) were identified from *C. gigas*. Their responses against acidification treatment during larval development stages were investigated to illustrate the impact of OA on the shell formation of oyster larvae by modulating the biogenesis of chitin.

Chitin synthase and chitinase were reported to be employed in many biological and physiological processes such as digestion (Mali et al., 2004), host defense (Boot et al., 1995, 2001), and most importantly, in biomineralization (Schonitzer and Weiss, 2007). In order to understand the modulation effect of chitin synthase and chitinase in shell formation, in the present study, a chitin synthase gene (*Cg*chs1) and a chitinase gene (*Cg*chit4) were identified from *C. gigas*. The length of *Cg*chs1 sequence was of 813 bp, encoding a putative peptide fragment of 271 amino acids with the theoretical molecular weight of 30.16 kDa. There was a Chitin_synth_2 domain in *Cg*chs1, which was the key domain to catalyze the chemical reaction (Sburlati and Cabib, 1986). Chitin synthases belong to the members of glycosyltransferases and consist of a transmembrane protein family with at least three transmembrane helices. The N- and C-terminal residues of chitin synthetase possibly differ even in the isoforms from the same species, but these two lower complexity motifs contain plenty of acidic or basic residues which are the main characteristics for various molluscan chitin synthase. In addition, the catalytic site of intracellular is highly conserved which possesses the function of catalytic chitin synthesis and plays a key role in the precisely controlled mineral deposition process of other mollusk as well as Pacific oyster C. gigas (Marin et al., 2008; Schonitzer et al., 2011). As for *Cg*chit4, the full length of cDNA sequence was of 2118 bp, encoding a putative polypeptide of 706 amino acids with the theoretical molecular weight of 80.34. A Glyco_18 domain was included in the *Cg*chit4, which was important for the hydrolysis of the glucosidic bond of carbohydrates (Renkema et al., 1995). *Cg*chs1 shared high sequence similarity (42–44%) with chitin synthase from other vertebrates and invertebrates. Phylogenic analysis also showed that *Cg*chs1 was close to chitin synthase from invertebrates such as *B. glabrata, M. yessoensis* (Bulawa, 1992; Luo et al., 2015). *Cg*chit4 exhibited high sequence similarity (41–52%) with chitinase from other vertebrates and invertebrates, and it was clustered with chitinases from invertebrates such as *P. fucata* and *H. cumingii* (Gao et al., 2014; Li et al., 2017). These results indicated that *Cg*chs1 and *Cg*chit4 might belong to the family of chitin synthase and chitinase in molluscs, respectively.

In order to investigate the possible function of *Cg*chs1 and *Cg*chit4 in shell formation of oyster larvae, their mRNA expression levels and localization were detected by quantitative real-time PCR and WMISH, and the larval morphological characteristics were observed by SEM. The expression level of *Cg*chs1 mRNA was extremely lower at D-shape larvae compared with that at trochophore and early D-shape larvae, while the expression level of *Cg*chit4 mRNA in early D-shape larvae was distinctly higher than that at trochophore and D-shape larvae. As shown in Figure 5, a uniformly calcified shell was successfully formed from trochophore to D-shape larvae at control group. Similar results have also been reported from other molluscan species. It was found that the chitin synthase could be detected in the shell margin of larval *M. galloprovincialis* which was the region involved in shell extend in early developmental stage (Weiss et al., 2006). Chitin synthase and chitinase are the key enzymes for chitin shell biosynthesis in molluscs. Chitin synthase is in charge of the synthesis of chitin, while chitinase is responsible for the degradation of chitin (Weiss and Schonitzer, 2006; Weiss et al., 2006). Results of this study suggested that synthesis of chitin was down-regulated and the degradation of chitin was prompted at the stages from trochophore to early D-shape larvae, which might cause the content reduction of chitin in the shell of oyster larvae. These results were in consistent with previous findings that biomineralization was activated from trochophore to D-shape larvae in molluscs, resulting in the organic-mineral composite (mainly CaCO_3_) shell layer to cover the chitin framework one.

According to previous study, there are several hypotheses trying to explain how shell formations in oyster larvae are affected by OA. Some researchers considered that OA lowered the calcium carbonate saturation state, which caused oyster larvae harder to form the complete calcified shell. It was also believed that acidification upset the acid-base balance between the marine organism and environment, and even the inner acid-base balance of organism (Zhao et al., 2017), which influenced the energy metabolism and homeostasis in oyster larvae (Lannig et al., 2010). We are inclined to a hypothesis that OA might disturb the biosynthesis of chitin and finally invite the failure of shell formation in oyster larvae. In the present study, the expression of *Cg*chs1 and *Cg*chit4, and the morphological characteristics of oyster larvae under acidification treatment were explored. It was found that in the moderate acidification treatment (pH 7.8) group, the expression level of *Cg*chs1 mRNA was decreased slightly in early D-shape stage, while that of *Cg*chit4 mRNA was obviously decreased in early D-shape stage in the severe acidification treatment (pH 7.4) group. These results suggested that the synthesis of chitin was significantly inhibited by severe acidification treatment (pH 7.4), while the degradation of chitin was dramatically activated. As shown in Figure 5, the shell formation in oyster larvae was indeed significantly suppressed by severe acidification treatment. Chitin is proverbial as a crucial component in molluscan shell and nacre biosynthesis. Chitin constructed the initial shell basis and supplied for other macromolecular and mineral deposition that visibly guide the biomineralization process, even in the system of crystal polymorphism (Levi-Kalisman et al., 2001; Zentz et al., 2001). Results in current research implied that the inhibition of biosynthesis of chitin caused by acidification conditions might be the reason for the failure of shell formation from trochophore to D-shape larvae during ontogenesis of oyster.

Moreover, the structural organization of chitin synthesizing complex in oyster larvae was further explored under normal and acidification conditions with WMISH. As shown in Figure 6, the *Cg*chs1 and *Cg*chit4 mRNA was mainly expressed in the beneath of newly formed shells. In trochophore stage, the positive signal was mainly located on the margin of shell field. In D-shape larvae stage, the positive signal was found on the edge of periostracum. These consequences were consistent with the anterior studies that chitin synthase could be detected in the shell gland region and related to shell biosynthesis of *M. galloprovincialis* larvae, and the inhibition of chitin synthase through Nikkomycin Z could dramatically alter the shell structure and function, for instance the bivalve hinge and the shell margin (Schönitzer and Weiss, 2007). Another previous study reported that the calcification taken place on the margin of the shell, among mantle tissue, periostracum and the shell itself (Marin et al., 2012). Therefore, all these results collectively suggested that the shell biosynthesis process and intricate framework compounds structure in oyster larvae were located close to the edges of shell. Acidification treatment could severely inhibit the biosynthesis of chitin, resulting in the failure of biomineralization in oyster larvae.

## Conclusion

In conclusion, a *Cg*chs1 and a *Cg*chit4 gene were identified from *C. gigas*, which belonged to chitin synthase and chitinase family, respectively. During the key developmental stages for shell formation, the chitin biosynthesis was decreased from trochophore to D-shape larvae since the down-regulated mRNA expression of *Cg*chs1, while the decomposition of chitin was accelerated due to the up-regulated mRNA expression level of *Cg*chit4. When oyster larvae suffered from CO_2_-induced acidification, the formation of calcified shell was severely inhibited, and the expression of *Cg*chs1 was significantly suppressed while that of *Cg*chit4 was dramatically activated, resulting in a decrease in chitin content on the edges of the shell. These results collectively suggested that OA might inhibit shell formation in oyster larvae by suppressing the biosynthesis of chitin.

## Data Availability

The raw data supporting the conclusions of this manuscript will be made available by the authors, without undue reservation, to any qualified researcher.

## Ethics Statement

This study was carried out in accordance with the recommendations of the Ethics Committee of Dalian Ocean University. The protocol was approved by the Ethics Committee of Dalian Ocean University.

## Author Contributions

YZ, ZL, XS, SH, and LW conceived and designed the experiments. YZ, ZL, and XS performed the experiments. YZ and ZL analyzed the data. LW and LS contributed reagents, materials, and analysis tools. YZ, ZL, LW, and LS wrote the manuscript. All authors read and approved the final manuscript.

## Conflict of Interest Statement

The authors declare that the research was conducted in the absence of any commercial or financial relationships that could be construed as a potential conflict of interest.

## Footnotes

1 http://www.ncbi.nlm.gov/blast

2 http://www.expasy.org

3 http://www.smart.emblheidelberg.de/

4 http://www.ebi.ac.uk/Tools/clustalw2/

5 http://www.biosoft.net/sms/index.html

## References

[B1] Barros P. Sobral P. Range P. Chícharo L. Matias D. (2013). Effects of sea-water acidification on fertilization and larval development of the oyster *Crassostrea gigas*. *J. Exp. Mar. Biolecol.* 440 200–206. 10.1016/j.jembe.2012.12.014

[B2] Barton A. Hales B. Waldbusser G. G. Langdon C. Feely R. A. (2012). The *Pacific oyster*, *Crassostrea gigas*, shows negative correlation to naturally elevated carbon dioxide levels: implications for near-term ocean acidification effects. *Limnol. Oceanogr.* 57 698–710. 10.4319/lo.2012.57.3.0698

[B3] Bevelander G. Nakahara H. (1969). An electron microscope study of the formation of the nacreous layer in the shell of certain bivalve molluscs. *Calcif. Tissue Res.* 3 84–92. 10.1007/bf02058648 5772447

[B4] Bielefeld U. Becker W. (1991). Embryonic development of the shell in biomphalaria glabrata (Say). *Int. J. Dev. Biol.* 35 121–131. 1768600

[B5] Boot R. G. Blommaart E. F. Swart E. Ghauharali-Van Der Vlugt K. Bijl N. Moe C. (2001). Identification of a novel acidic mammalian chitinase distinct from chitotriosidase. *J. Biol. Chem.* 276 6770–6778. 10.1074/jbc.m009886200 11085997

[B6] Boot R. G. Renkema G. H. Strijland A. Van Zonneveld A. J. Aerts J. M. (1995). Cloning of a cDNA encoding chitotriosidase, a human chitinase produced by macrophages. *J. Biol. Chem.* 270 26252–26256. 10.1074/jbc.270.44.26252 7592832

[B7] Bulawa C. E. (1992). CSD2, CSD3, and CSD4, genes required for chitin synthesis in *Saccharomyces cerevisiae*: the CSD2 gene product is related to chitin synthases and to developmentally regulated proteins in Rhizobium species and Xenopus laevis. *Mol. Cell Biol.* 12 1764–1776. 10.1128/mcb.12.4.1764 1532231 PMC369620

[B8] Carriker M. R. Palmer R. E. (1979). Ultrastructural morphogenesis of prodissoconch and early dissoconch valves of the oyster *Crassostrea virginica*. *Proc. Natl. Shellfish Assoc.* 69 103–128.

[B9] Eyster L. S. (1986). Shell inorganic composition and onset of shell mineralization during bivalve and gastropod embryogenesis. *Biol. Bull.* 170 211–231. 10.2307/1541804

[B10] Eyster L. S. Morse M. P. (1984). Early shell formation during molluscan embryogenesis, with new studies on the surf clam. Spisula-Solidissima. *Am. Zool.* 24 871–882. 10.1093/icb/24.4.871

[B11] Gao L. Xu G. J. Su H. Gao X. G. Li Y. F. Bao X. B. (2014). Identification and expression analysis of cDNA encoding chitinase-like protein (CLP) gene in Japanese scallop *Mizuhopecten yessoensis*. *Genet. Mol. Res.* 13 10727–10740. 10.4238/2014.December.18.14 25526193

[B12] Gazeau F. Gattuso J. P. Dawber C. Pronker A. E. Peene F. Peene J. (2010). Effect of ocean acidification on the early life stages of the blue mussel *Mytilus edulis*. *Biogeosciences* 7 2051–2060. 10.1080/15287394.2011.550460 21391089

[B13] Hendriks I. E. Duarte C. M. Olsen Y. S. Steckbauer A. Ramajo L. Moore T. S. (2015). Biological mechanisms supporting adaptation to ocean acidification in coastal ecosystems. *Estuar. Coast. Shelf. S* 152 A1–A8.

[B14] Kerr R. A. (2010). Ocean acidification unprecedented, unsettling. *Science* 328 1500–1501. 10.1126/science.328.5985.1500 20558701

[B15] Kin K. Kakoi S. Wada H. (2009). A novel role for dpp in the shaping of bivalve shells revealed in a conserved molluscan developmental program. *Dev. Biol.* 329 152–166. 10.1016/j.ydbio.2009.01.021 19382296

[B16] Kniprath E. (1979). Functional-morphology of the embryonic shell-gland in the conchiferous mollusks. *Malacologia* 18 549–552.

[B17] Kniprath E. (1981). Ontogeny of the molluscan shell field - a review. *Zool. Scr.* 10 61–79. 10.1111/j.1463-6409.1981.tb00485.x

[B18] Kroeker K. J. Kordas R. L. Crim R. N. Singh G. G. (2010). Meta-analysis reveals negative yet variable effects of ocean acidification on marine organisms. *Ecol. Lett.* 13 1419–1434. 10.1111/j.1461-0248.2010.01518.x 20958904

[B19] Kurihara H. Kato S. Ishimatsu A. (2007). Effects of increased seawater pCO2 on early development of the oyster *Crassostrea gigas*. *Aquat. Biol.* 1 91–98. 10.3354/ab00009

[B20] Lannig G. Eilers S. Pörtner H. O. Sokolova I. M. Bock C. (2010). Impact of ocean acidification on energy metabolism of oyster, *Crassostrea gigas* - changes in metabolic pathways and thermal response. *Mar. Drugs* 8 2318–2339. 10.3390/md8082318 PMC2953406 20948910

[B21] Levi-Kalisman Y. Falini G. Addadi L. Weiner S. (2001). Structure of the nacreous organic matrix of a bivalve mollusk shell examined in the hydrated state using cryo-TEM. *J. Struct. Biol.* 135 8–17. 10.1006/jsbi.2001.4372 11562161

[B22] Li H. Wang D. Deng Z. Huang G. Fan S. Zhou D. (2017). Molecular characterization and expression analysis of chitinase from the pearl oyster Pinctada fucata. *Comp. Biochem. Physiol. B Biochem. Mol. Biol.* 203 141–148. 10.1016/j.cbpb.2016.10.007 27826036

[B23] Liu Z. Wang L. Yan Y. Zheng Y. Ge W. Li M. (2018). D1 dopamine receptor is involved in shell formation in larvae of *Pacific oyster Crassostrea gigas*. *Dev. Comp. Immunol.* 84 337–342. 10.1016/j.dci.2018.03.009 29550270

[B24] Livak K. J. Schmittgen T. D. (2001). Analysis of relative gene expression data using real-time quantitative PCR and the 2- ΔΔCT method. *Methods* 25 402–408. 10.1006/meth.2001.1262 11846609

[B25] Luo Y. J. Takeuchi T. Koyanagi R. Yamada L. Kanda M. Khalturina M. (2015). The Lingula genome provides insights into brachiopod evolution and the origin of phosphate biomineralization. *Nat. Commun.* 6:8301. 10.1038/ncomms9301 26383154 PMC4595640

[B26] Mali B. Mohrlen F. Frohme M. Frank U. (2004). A putative double role of a chitinase in a cnidarian: pattern formation and immunity. *Dev. Comp. Immunol.* 28 973–981. 10.1016/j.dci.2004.04.002 15236928

[B27] Marin F. Le Roy N. Marie B. (2012). The formation and mineralization of mollusk shell. *Front. Biosci.* 4:1099–1125. 10.2741/s321 22202112

[B28] Marin F. Luquet G. Marie B. Medakovic D. (2008). Molluscan shell proteins: primary structure, origin, and evolution. *Curr. Top. Dev. Biol.* 80 209–276. 10.1016/s0070-2153(07)80006-8 17950376

[B29] McDonald M. R. Mcclintock J. B. Amsler C. D. Rittschof D. Angus R. A. Orihuela B. (2009). Effects of ocean acidification over the life history of the barnacle *Amphibalanus amphitrite*. *Mar. Ecil. Prog. Ser.* 385 179–187. 10.3354/meps08099

[B30] Miller A. W. Reynolds A. C. Sobrino C. Riedel G. F. (2009). Shellfish face uncertain future in high CO2 world: influence of acidification on oyster larvae calcification and growth in estuaries. *PLoS One* 4:e5661. 10.1371/journal.pone.0005661 19478855 PMC2682561

[B31] Nudelman F. Gotliv B. A. Addadi L. Weiner S. (2006). Mollusk shell formation: mapping the distribution of organic matrix components underlying a single aragonitic tablet in nacre. *J. Struct. Biol.* 153 176–187. 10.1016/j.jsb.2005.09.009 16413789

[B32] Orr J. C. Fabry V. J. Aumont O. Bopp L. Doney S. C. Feely R. A. (2005). Anthropogenic ocean acidification over the twenty-first century and its impact on calcifying organisms. *Nature* 437 681–686. 10.1038/nature04095 16193043

[B33] Parker L. M. Ross P. M. O’connor W. A. Borysko L. Raftos D. A. Pörtner H. (2012). Adult exposure influences offspring response to ocean acidification in oysters. *Global. Change. Biol.* 18 82–92. 10.1111/j.1365-2486.2011.02520.x

[B34] Peters W. (1972). Occurence of chitin in mollusca. *Biochem. Bioph. Res. Co.* 41 541–550. 10.1016/0305-0491(72)90117-4

[B35] Renkema G. H. Boot R. G. Muijsers A. O. Donker-Koopman W. E. Aerts J. M. (1995). Purification and characterization of human chitotriosidase, a novel member of the chitinase family of proteins. *J. Biol. Chem.* 270 2198–2202. 10.1074/jbc.270.5.2198 7836450

[B36] Sabine C. L. Feely R. A. Gruber N. Key R. M. Lee K. Bullister J. L. (2004). The oceanic sink for anthropogenic CO2. *Science* 305 367–371. 10.1126/science.1097403 15256665

[B37] Sburlati A. Cabib E. (1986). Chitin synthetase 2, a presumptive participant in septum formation in Saccharomyces cerevisiae. *J. Biol. Chem.* 261 15147–15152. 2945823

[B38] Schonitzer V. Eichner N. Clausen-Schaumann H. Weiss I. M. (2011). Transmembrane myosin chitin synthase involved in mollusc shell formation produced in dictyostelium is active. *Biochem. Biophys. Res. Commun.* 415 586–590. 10.1016/j.bbrc.2011.10.109 22079092

[B39] Schonitzer V. Weiss I. M. (2007). The structure of mollusc larval shells formed in the presence of the chitin synthase inhibitor nikkomycin Z. *BMC Struct. Biol.* 7:71. 10.1186/1472-6807-7-71 17986326 PMC2241824

[B40] Schönitzer V. Weiss I. M. (2007). The structure of mollusc larval shells formed in the presence of the chitin synthase inhibitor nikkomycin Z. *BMC Struct. Biol.* 7:71. 10.1186/1472-6807-7-71 17986326 PMC2241824

[B41] Talmage S. C. Gobler C. J. (2009). The effects of elevated carbon dioxide concentrations on the metamorphosis, size, and survival of larval hard clams (*Mercenaria mercenaria*), bay scallops (*Argopecten irradians*), and Eastern oysters (*Crassostrea virginica*). *Limno. Oceanogr.* 54 2072–2080. 10.4319/lo.2009.54.6.2072

[B42] Talmage S. C. Gobler C. J. (2010). Effects of past, present, and future ocean carbon dioxide concentrations on the growth and survival of larval shellfish. *Proc. Natl. Acad. Sci. U.S.A.* 107 17246–17251. 10.1073/pnas.0913804107 20855590 PMC2951451

[B43] Thisse C. Thisse B. (2008). High-resolution in situ hybridization to whole-mount zebrafish embryos. *Nat. Protoc.* 3 59–69. 10.1038/nprot.2007.514 18193022

[B44] Waldbusser G. G. Brunner E. L. Haley B. A. Hales B. Langdon C. J. Prahl F. G. (2013). A developmental and energetic basis linking larval oyster shell formation to acidification sensitivity. *Geophys. Res. Lett.* 40 2171–2176. 10.1002/grl.50449

[B45] Waldbusser G. G. Hales B. Langdon C. J. Haley B. A. Schrader P. Brunner E. L. (2014). Saturation-state sensitivity of marine bivalve larvae to ocean acidification. *Nat. Clim. Chang.* 5 273–280. 10.1038/nclimate2479

[B46] Waldbusser G. G. Salisbury J. E. (2014). Ocean acidification in the coastal zone from an organism’s perspective: multiple system parameters, frequency domains, and habitats. *Ann. Rev. Mar. Sci.* 6 221–247. 10.1146/annurev-marine-121211-172238 23987912

[B47] Waller T. R. (1981). Functional morphology and development of veliger larvae of the European oyster, *Ostrea edulis* Linne. *Smithsonian. Contrib. Zool.* 328 1–70. 10.5479/si.00810282.328

[B48] Weiss I. M. Schonitzer V. (2006). The distribution of chitin in larval shells of the bivalve mollusk *Mytilus galloprovincialis*. *J. Struct. Biol.* 153 264–277. 10.1016/j.jsb.2005.11.006 16406681

[B49] Weiss I. M. Schonitzer V. Eichner N. Sumper M. (2006). The chitin synthase involved in marine bivalve mollusk shell formation contains a myosin domain. *FEBS Lett.* 580 1846–1852. 10.1016/j.febslet.2006.02.044 16513115

[B50] Weiss I. M. Tuross N. Addadi L. Weiner S. (2002). Mollusc larval shell formation: amorphous calcium carbonate is a precursor phase for aragonite. *J. Exp. Zool.* 293 478–491. 10.1002/jez.90004 12486808

[B51] Zentz F. Bedouet L. Almeida M. J. Milet C. Lopez E. Giraud M. (2001). Characterization and quantification of chitosan extracted from nacre of the abalone Haliotis tuberculata and the oyster *Pinctada maxima*. *Mar. Biotechnol.* 3 36–44. 10.1007/s101260000042 14961388

[B52] Zhang G. Fang X. Guo X. Li L. Luo R. Xu F. (2012). The oyster genome reveals stress adaptation and complexity of shell formation. *Nature* 490 49–54. 10.1038/nature11413 22992520

[B53] Zhang H. Song L. Li C. Zhao J. Wang H. Qiu L. (2008). A novel C1q-domain-containing protein from Zhikong scallop Chlamys farreri with lipopolysaccharide binding activity. *Fish. Shellfish Immunol.* 25 281–289. 10.1016/j.fsi.2008.06.003 18603000

[B54] Zhao X. Shi W. Han Y. Liu S. Guo C. Fu W. (2017). Ocean acidification adversely influences metabolism, extracellular pH and calcification of an economically important marine bivalve. Tegillarca granosa. *Mar. Environ. Res.* 125 82–89. 10.1016/j.marenvres.2017.01.007 28188988

[B55] Zhou Z. Wang L. Shi X. Yue F. Wang M. Zhang H. (2012). The expression of dopa decarboxylase and dopamine beta hydroxylase and their responding to bacterial challenge during the ontogenesis of scallop Chlamys farreri. *Fish. Shellfish Immunol.* 33 67–74. 10.1016/j.fsi.2012.04.002 22521420

